# Electronic Nicotine Delivery System: End to Smoking or Just a New Fancy Cigarette

**DOI:** 10.7759/cureus.43425

**Published:** 2023-08-13

**Authors:** Samrudhi S Kotewar, Aayushi Pakhale, Rupali Tiwari, Amit Reche, Shriya R Singi

**Affiliations:** 1 Department of Public Health Dentistry, Sharad Pawar Dental College ad Hospital, Datta Meghe Institute of Higher Education and Research (Deemed to be University), Wardha, IND; 2 Department of Oral Pathology and Microbiology, Sharad Pawar Dental College and Hospital, Datta Meghe Institute of Higher Education and Research (Deemed to be University), Wardha, IND; 3 Department of Public Health Dentistry, Sharad Pawar Dental College and Hospital, Datta Meghe Institute of Higher Education and Research (Deemed to be University), Wardha, IND; 4 Department of Research and Development, Jawaharlal Nehru Medical College, Datta Meghe Institute of Higher Education and Research (Deemed to be University), Wardha, IND

**Keywords:** electronic nicotine delivery systems, heat-not-burn, personal vaporizers, vaping, nicotine, e-cigarettes

## Abstract

Smoking and tobacco chewing are the predominant causes of oral cancer. Tobacco is the second-most widely consumed psychoactive substance. There are numerous ways to quit smoking, of which one is electronic cigarettes (e-cigarettes). E-cigarette use is a brand-new, global trend. E-cigarette is a battery-operated device that heats a liquid to create a vapor that the consumer inhales. Several countries have acknowledged that the first step toward electronic nicotine delivery system (ENDS) management is a precise classification of ENDS within the limits of current legislation. Countries have currently categorized ENDS into four generations. People's perceptions about tobacco products have altered recently as a consequence of the advertising of ENDS. The likelihood of starting to smoke cigarettes was four times higher in adolescents who used ENDS, and the probability of quitting was reduced and often prolonged in those who used ENDS. In addition, ENDS normalizes smoking-like actions including inhaling in and exhaling smoke. Adverse marketing via geographic locations and social media platforms, as well as nicotine's irreversible effects on growing adolescent and young adult brains that predispose individuals to addicted behaviors, may be responsible for their rising appeal among teenagers. Despite this, ENDS use has risen among young individuals who have never smoked and undoubtedly face more health risks than those who do not use ENDS. The oral cavity is the first to encounter ENDS in individuals and where it initially affects the human system. As a known contributor to cardiovascular diseases, neurological conditions, and cancers, nicotine seems to be a serious cause for concern. This review provides a concise summary of the research on the components, mode of action, applications, and effects of e-cigarettes on oral as well as systemic systems.

## Introduction and background

The World Health Organization (WHO) estimates that cancer is a leading cause of death worldwide, accounting for nearly 10 million deaths in 2020, or almost one in six deaths [1]. Around 5 million people globally die annually from smoking-related causes, accounting for 12% of all adult fatalities [2]. There is evidence that smoking causes cancer and raises the risk of other smoking-related diseases, including coronary heart disease, cancer recurrence, second primary tumors, and death if a person continues to smoke after being diagnosed with cancer [3].

The majority of smokers who are identified with head and neck cancer try to quit, but a sizable percentage eventually fail at some point [4,5]. However, more than 80% of smokers try to give up on their revert during the initial month of withdrawal, and only around 3% can quit after six months. This demonstrates the severity of tobacco addiction and the chronic aspect of the condition [6]. The highly addictive component of tobacco, nicotine, keeps people smoking even when they have health issues or want to stop. However, the adverse effects of smoking tobacco products are not solely attributable to nicotine [7]. According to research, nicotine delivered alone in various tobacco cessation regimens is safe and effective as a smoking cessation aid.

Novel nicotine delivery technologies, known as electronic nicotine delivery systems (ENDS) or e-cigarettes, have also evolved, with the ability to minimize the adverse consequences of tobacco smoking among individuals who switched entirely from combustible to e-cigarettes [8,9]. Nicotine patches, lozenges, mouth sprays, inhalers, nicotine gum, and electronic nicotine cigarettes are some ways to quit smoking.

## Review

Search methodology

The search strategy involved searching Google Scholar, Scopus, and PubMed databases for published articles relevant to the topic. The research included quantitative and qualitative research studies and academic papers to ensure a thorough understanding of the subject matter. The search involved the use of specific keywords such as "electronic nicotine delivery systems," "e-cigarettes," "nicotine," and "vaping." The studies that focused on ENDS were prioritized. In addition, preference was given to studies that were published in reputable sources, peer-reviewed journals, and conference proceedings, and were published recently to ensure that recent research is considered. The articles that were not published in English and showed the non-availability of the full text were excluded. The review confirmed the inclusion of high-quality studies that addressed the study's objectives by adhering to these selection criteria.

What are electronic cigarettes?

The introduction of vaping devices has transformed people's perceptions of tobacco products. [2]. Electronic cigarettes (E-cigarettes) were first launched in China in 2003 and made their way to the U.S. industry in 2007 [10-12]. The term “electronic nicotine delivery systems,” which refers to the various varieties of nicotine-containing e-cigarette devices, was first used by WHO in 2009 [13]. These terms describe a battery-powered device that uses electrical heating to convert a solution (also called "e-juice") containing nicotine, propylene glycol, and flavors into a misty vapor that is inhaled by the user (also known as the "vaper") to simulate cigarette smoke [14]. Interestingly, there is no combustion or smoke from tobacco involved with vaping. Since e-cigarettes do not burn tobacco, users do not expose themselves or someone else to several harmful components and particles that traditional cigarettes release in their smoke [15]. The Internet has enabled e-cigarettes to be readily accessible worldwide through the Internet and online shopping. However, some experts have expressed concerns that the popularity of e-cigarettes among young people owing to their assumed low risk may serve as a bridge to future smoking, and regular use may hinder users from quitting by prolonging their dependence on nicotine [16,17].

Types of electronic nicotine delivery systems

Several countries have acknowledged that the first step toward ENDS management is a precise classification of ENDS within the limits of current legislation. Countries have currently categorized ENDS into the following four generations.

First-generation e-cigarettes, sometimes known as "cig-a-likes," featured fixed and low-voltage electrodes and mimicked traditional cigarettes' appearance and feel. There are three variants of the atomizing devices from the first generation of cig. The original e-cigarettes' first version is a three-piece type comprising a unique atomizing device, battery, and fluid tank [18]. Currently, there is no supply of original classic-style e-cigarettes. The second variant is a two-piece design, combining atomizing units, a fluid reservoir, and a distinct battery. The third version is a one-piece disposable that was launched in 2013. It integrates the atomizing device, fluid tank, and battery into one component [18-20]. Second-generation e-cigarettes: larger variable voltage batteries, usually described as "pen-style batteries," are frequently seen in second-generation e-cigarettes, also referred to as "cartomizers" [20-22]. Clearomizers of the second generation include a separable atomizing unit with a filament inside a shell that screws into the fluid tank and the battery. Compared to e-cigarettes made in the cig-a-like fashion, clearomizers are translucent and feature larger fluid reservoirs (or tanks). Any commercially marketed refill fluids may fill clearomizers [23]. Third-generation electric cigarettes are referred to as "Mods" because they involve modified batteries that let users adjust the voltage, wattage, and power. Some variants offer additional functions, such as charging a cell phone [23]. The third generation has three types of atomizing units: sub-ohm, replaceable dripping, and various styles [24]. Different forms and coil configurations may be found in these atomizing devices. Typically, the fluid tanks may be detached for further customization and could be more significant than clearomizers. The fourth generation of e-cigarettes consists of the pod-style with a fixed voltage and different-shaped batteries, including USB or teardrop-shaped ones. There are many new members of this generation, which is quickly evolving [24-26].

Components and mechanism of action of ENDS

**Figure 1:**
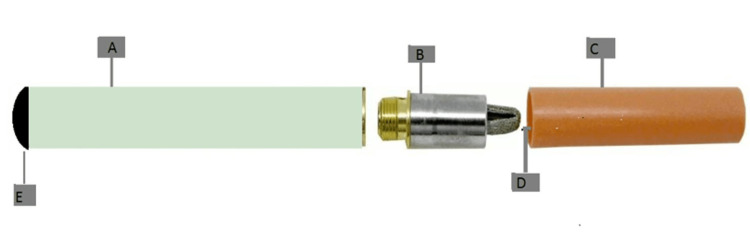
Components of electronic nicotine delivery systems A, battery component; B, atomizing device; C, inhaler; D, liquid container; E, indicator light Note: the figure was created by the author.

The components of an e-cigarette are as follows: cartridge(s) (CA) containing a nicotine solution dissolved in propylene glycol or glycerin; thermal element to vaporize the nicotine solution; micro-controller equipped with a detector that activates the heating element whenever the e-cigarette is inhaled; rechargeable battery; and, sporadically, an LED that imitates the glow of a burning cigarette nozzle [27]. These components are depicted in Figure 1.

ENDS are distinct from traditional tobacco products such that they generate an inhalable aerosol using a vaporization device, sometimes known as a "vape" [28]. Most ENDS are fashioned to mimic conventional tobacco cigarettes. The more current designs frequently offer user control over voltage, coil type modification, wicking material, and reservoir size. These factors can be altered to provide distinct heating patterns that affect the aerosol's composition. The aerosolized liquid that the consumer inhales (e-liquid) comes into direct contact with the vaporizer's parts. After administration of electric current plus intense heating, components from the vaporizer also have been discovered in the inhaled aerosol [28-30]. Propylene glycol, vegetable glycerin, or sometimes both may be used as the base solvent in e-liquid preparations in addition to NICOTINE. One or more flavored ingredients are also frequently incorporated. There is an overrun of flavoring goods on the market. Around 7,764 different flavors were reportedly offered by 466 companies in 2014 [31]. Some compounds have drawn interest due to their acute toxicology or addiction-inducing qualities, making them far from being non-intrusive [32].

Advantages and disadvantages of ENDS

Many ENDS are fashioned to mimic conventional tobacco cigarettes. They heat and evaporate a nicotine-containing solution. Because they create fewer toxins in the vapor that is provided to the user, several proponents of tobacco prevention approaches have emphasized these devices as possible alternatives to cigarettes [2]. Inadequate data on ingredients and emissions, particularly with long-term use, and unsubstantiated commercial claims, such as a smoking cessation tool, offer causes for worry, nevertheless. But lately, ENDS has been the subject of a controversial discussion. On the one side, ENDS are viewed as a substitute for cigarettes and an aid to cigarette smokers in ongoing efforts to give up tobacco use; however, on the other side, there are growing questions about the risk to public health associated with ENDS use [2]. A practical comparison between the advantages and disadvantages of using ENDS is presented in Table 1.

**Table 1:** Advantages and disadvantages of ENDS ENDS, electronic nicotine delivery systems

Advantages of ENDS	Disadvantages of ENDS
Substitutes to smoking cigarettes	There are few statistics on public health hazard
Relative to conventional cigarettes, there are reduced rates of volatile compounds and nitrosamine in ENDS	It serves as a gateway for the consumption of various nicotine substances, including traditional cigarettes, among teenage consumers
Aid in the goal of quitting smoking	Risk of developing a dual smoking habit
Minimize the environmental impact of tobacco smoking and cigarette butts	Throwaway plastic cartilage and removable coils are harmful to the environment
Switching toward electronic cigarettes can lower the health hazards for existing tobacco smokers	Due to a lack of safety protocols, electronic cigarette liquid poses a danger of overdose. Due to battery overheating and potential explosion, electronic cigarette mods provide a risk for harm.
Flavoring, additives, and nicotine are all adjustable and optional	Nicotine intake is much more effective than in traditional cigarettes, raising the risk of dependency. Excessive consumption among first-time users exposes a new, mostly teenage group to the possibility of nicotine dependence.

Effects of ENDS on the oral cavity

Early studies revealed that ENDS transmit toxins typically related to tobacco-related illness at lower levels than cigarettes. The oral cavity is the first contact point for ENDS and the earliest affected structure in individuals [33]. Electronic nicotine delivery devices (often called e-cigarettes) are a popular modern tobacco product, especially among younger generations. Yet, they remain freely available via the Internet and at retail stores. Slight oral complaints associated with ENDS consumers involved reduced salivation, causing dryness and mouth and throat discomfort. Additional undesirable consequences included enhanced inflammatory reaction and cell differentiation, impaired oral wound repair, and masked periodontal disease symptoms such as bleeding on probing [34]. E-cigarette users reported toothache at a rate of 6.9% to 8.1%, while traditional cigarette smokers reported it at 3.9% to 16.7% [35,36]. Cracked or broken teeth were more common among e-cigarette consumers than non-smokers [35]. Toothache has been reported by 6.9-8.1% of e-cigarette consumers and 3.9-16.7% of traditional cigarette smokers [36]. Tooth abscess was reported by 2.4% of smokers who switched to e-cigarette use, and hypersensitivity was reported by 29.1% of people who used to smoke e-cigarettes, owing to the flavoring and viscosity of e-liquids, which may cause enamel erosion and strengthen cariogenic microorganisms. Compared to an unflavored E-liquid, the cariogenic ability of flavors increased biofilms' production, and the E-liquid's viscosity aided in the attachment of Streptococcus mutans toward the tooth surface [37]. There are several factors that may contribute to e-cigarette-related tooth decay. One of these factors is the aerosols emitted by e-cigarettes, which can cause Streptococcus mutans to adhere to the tooth surfaces. This has been linked to the development of pit and fissure caries [38]. These aerosols contain acetic acid, lactic acid, and propionaldehyde, which can cause demineralization of enamel [39]. In addition, specific types of e-cigarettes contain high levels of caries-causing sugars such as fructose and sucrose [40]. Furthermore, research has shown that vaping can result in xerostomia, a condition that promotes the growth of dental caries [41].

Overall adverse effect of ENDS on consumers

Nicotine represents significant concern as a proven component of cardiovascular ailments, neurological disorders, and malignancies [42]. Using ENDS increases systolic and diastolic pressure irrespective of nicotine concentration [43]. A characteristic of ENDS exposure that is not found after smoking and seems nicotine-independent is the promotion of lipid deposition in alveolar macrophages [44].

Cardiovascular System

When combined with cigarettes, the risk of cardiovascular disease, myocardial infarction (MI), and stroke rise by 36% when compared to cigarette use alone [45]. Other factors, such as pulse rate and sympathetic dominance, seem to be affected by smoking. Within 14 days, smokers who switched to vaping had lower carboxyhemoglobin concentrations [46]. Additionally, a review [47] highlights that the aerosols in ENDS contain carbonyls in levels that can be harmful to the cardiovascular health and also deliver nicotine, which can potentially elevate the chances of cardiovascular disease.

Airway Inflammation and Injury

The airway mucosa of healthy e-cigarette users has been seen to be erythematous and irritable, and cases of more severe bronchial damage have been documented [48]. MUC5AC mucin levels were shown to be elevated in both bronchial epithelia and airway secretions, despite the fact that many of those who used e-cigarette users were smokers previously [49]. Additionally, proteomics of sputum from e-cigarette users revealed increased neutrophil being activated, involving myeloperoxidase and neutrophil elastase, along with proteinase-3 [50]. A rise in protease concentrations was previously found in the lungs of tobacco smoker’s lungs [51], and the link between smoking, elevated proteolysis, and damage to the lungs is causal, implying that the protease concentrations have an additional biomarker that could be beneficial in investigating the effects of e-cigarettes on the lung [52].

Effects on Immunity

Nasal scrape biopsies comparing smokers, non-smokers, and vapers revealed significant immunosuppression with e-cigarette use at the gene level [53]. E-cigarette aerosol was inhaled by healthy non-smokers, and bronchoalveolar lavage was collected to examine alveolar macrophages [54]. More than 60 genes' expression was changed in e-cigarette users. Neutrophil extracellular trap (NET) creation, also known as NETosis, is an inherent defense mechanism in which neutrophils lyse DNA and then release it into the extracellular space to aid in the immobilization of bacteria, a process that may adversely affect the lung [55]. Neutrophils from longtime vapers have been discovered to be more likely to generate NETs compared to those via cigarette smokers or non-smokers [50]. Given that e-cigarettes may decrease neutrophil phagocytosis, [56] these findings suggest that e-cigarette users' neutrophil function may be affected.

## Conclusions

Cigarette smoking is one of the frequently used methods of nicotine consumption. Side effects of smoking are now well recognized by consumers, and they still manage to find a new way of consumption. ENDS was manufactured as an anti-nicotine or anti-smoking assistance but unfortunately became a smoking encouragement. No ENDS have been granted FDA approval for smoking cessation yet, and ENDS use should not be justified as the lesser of two evils. Its packaging has been modified fancily to make it attractive and is readily available online. However, the recurrence of smoking, ongoing dual use of e-cigarettes, and the possibility that non-smokers using e-cigarettes may raise their risk of starting to smoke traditional combustible cigarettes are serious concerns. E-cigarettes contain a noticeable amount of harmful ingredients, but still at lower levels than traditional cigarettes. An overall risk evaluation seems challenging because there are so many different kinds of ENDS, and various electronic cigarettes emit different amounts of toxic substances. ENDS was designed to end smoking, but it is now upholding a new era of smoking.
